# Neuroimmunology of Common Parasitic Infections in Africa

**DOI:** 10.3389/fimmu.2022.791488

**Published:** 2022-02-10

**Authors:** Richard Idro, Rodney Ogwang, Antonio Barragan, Joseph Valentino Raimondo, Willias Masocha

**Affiliations:** ^1^ College of Health Sciences, Makerere University, Kampala, Uganda; ^2^ Centre of Tropical Neuroscience, Kitgum, Uganda; ^3^ Nuffield Department of Medicine, Centre for Tropical Medicine and Global Health, University of Oxford, Oxford, United Kingdom; ^4^ Kenya Medical Research Institute (KEMRI) - Wellcome Trust Research Programme, Nairobi, Kenya; ^5^ Department of Molecular Biosciences, The Wenner-Gren Institute, Stockholm University, Stockholm, Sweden; ^6^ Division of Cell Biology, Department of Human Biology, Neuroscience Institute and Institute of Infectious Disease and Molecular Medicine, Faculty of Health Sciences, University of Cape Town, Cape Town, South Africa; ^7^ Department of Pharmacology and Therapeutics, Faculty of Pharmacy, Kuwait University, Safat, Kuwait

**Keywords:** brain disorders, *Plasmodium falciparum*, *Trypanosoma brucei* spp., *Toxoplasma gondii*, *Taenia solium*, neuro-infections, immune system, glia

## Abstract

Parasitic infections of the central nervous system are an important cause of morbidity and mortality in Africa. The neurological, cognitive, and psychiatric sequelae of these infections result from a complex interplay between the parasites and the host inflammatory response. Here we review some of the diseases caused by selected parasitic organisms known to infect the nervous system including *Plasmodium falciparum*, *Toxoplasma gondii*, *Trypanosoma brucei* spp., and *Taenia solium* species. For each parasite, we describe the geographical distribution, prevalence, life cycle, and typical clinical symptoms of infection and pathogenesis. We pay particular attention to how the parasites infect the brain and the interaction between each organism and the host immune system. We describe how an understanding of these processes may guide optimal diagnostic and therapeutic strategies to treat these disorders. Finally, we highlight current gaps in our understanding of disease pathophysiology and call for increased interrogation of these often-neglected disorders of the nervous system.

## Introduction

Some of the most extraordinary parasites are those that manage to establish infection in the human central nervous system (CNS). Because parasitic infections likely remain more prevalent in Africa than in any other continent, knowledge on their diverse CNS manifestations is of outmost importance. Adding to the death toll caused by cerebral parasitic infections, the neurological, cognitive, or mental health problems affect millions of Africans annually. Despite this, precise estimates of morbidity are lacking, and knowledge remains limited on the pathogenesis of CNS injury for many of these diseases. Prevention and control of these parasitic CNS infections therefore, remains a global research priority (1).

Parasites as infectious agents are a diverse group of unicellular (e.g., protozoa) and multicellular (e.g., helminths) organisms with complex life cycles and a variety of hosts, including humans. The geographical distribution and transmission dynamics of parasitic infections are often dictated by the ecosystem, with presence of specific insect vectors, zoonotic transmission, and reservoirs. Consequently, they are often locally endemic, and their prevalence depends on a variety of factors, including socio-economic factors (2).

The human brain is protected by several cellular barriers that regulate or restrict passage of molecules and cells, including microorganisms, to the brain parenchyma. Three main barrier systems protect neurons from blood-borne external insults, such as infection: the blood-brain barrier (BBB), the blood-cerebrospinal fluid barrier (BCSFB) and the meningeal barriers (3, 4). Inflammation can cause dysfunction of these barrier systems. While brain-resident immune cells and infiltrating leukocytes are central to limit infections by parasites that successfully translocate, the inflammatory responses may also severely damage or alter neuronal function (5). Thus, protective effects and detrimental inflammatory responses need to be balanced to minimize injury (6).

Being eukaryotic organisms, protozoa and helminths represent a particular challenge to the immune system. This is explained partly by their elaborate immune evasion mechanisms. Moreover, parasites undergo complex life cycles comprised sometimes of antigenically distinct extracellular and obligate intracellular stages. As motile organisms, parasites have also developed diverse strategies to migrate or be transported in tissues, resulting in systemic dissemination in the human body. These include mechanisms for translocation across the BBB. The broad array of immune evasion mechanisms and versatility of the host-pathogen interplay is possibly best illustrated by the diverse clinical presentations. While some parasites can cause acute life-threatening neurological damage, their adaptation to the human host also permits chronic, sometimes life-long, CNS infection (7–9).

Thus, to establish an infection in the CNS, a parasite must first breach the normally non-permissive cellular barriers of the brain and then be able to evade the immune responses unique to the CNS. The clinical CNS manifestations are often associated to the processes that result from the specific host-parasite interplay, which remains only partly understood. Here, we outline the current knowledge on host-pathogen interactions and neuro-immunopathogenesis for selected clinically relevant CNS infections by parasites in Africa. The parasites covered in depth, *Plasmodium* spp.*, Trypanosoma brucei* spp.*, Toxoplasma gondii* and *Taenia solium*, were chosen as examples of parasitic infections implicated in neurological disorders, such as epilepsy, sleeping sickness, headaches and cognitive impairment. Other endemic parasites, amoebas, *Echinococcus* spp., *Onchocerca* spp., *Paragonimus* spp., *Schistosoma* spp., *Sparganosis* spp., and *Toxocara* spp., that present with neurological manifestations are also covered.

## Neuro-Immunology of Cerebral Malaria

### Pathogen Description, Prevalence in Africa, Signs, and Symptoms

#### Burden and Transmission

Malaria is a mosquito borne disease and a leading cause of ill health and death especially among children in sub-Saharan Africa. In 2019, there were an estimated 229 million cases and 409 000 deaths globally. Five African countries - Nigeria (27%), the Democratic Republic of the Congo (12%), Uganda (5%), Mozambique (4%) and Niger (3%) accounted for over 50% of the deaths (10). Human disease is caused by four species of the genus *Plasmodium*: *Plasmodium falciparum, vivax, ovale* and *malariae.* There have also been outbreaks of infections by the monkey parasite – *Plasmodium knowelsi* in Southeast Asia. *Plasmodium falciparum* remains the most prevalent agent. It also causes the most severe infections. In contrast, by 2019, the proportion of clinical cases caused by *Plasmodium vivax* had reduced to 3% from about 7% in the year 2000 (10).

#### Life Cycle

Malaria parasites are transmitted by female anopheline mosquitoes and although over 100 species can transmit the parasite, in Africa, transmission is largely by *Anopheles arabiensis*, *Anopheles coluzzii* and *Anopheles gambiae* from the Gambiae complex and *Anopheles funestus* from the Funestus subgroup (11).

The parasite’s life cycle is made of a vector and human exoerythrocytic (hepatic) and erythrocytic stages. During a blood meal, the female mosquito (vector) bites and injects mature sporozoites from its salivary glands into the host’s circulation. These quickly invade the liver hepatocytes and start asexual reproduction and multiplication as in tissue schizogony (exoerythrocytic stage). The tissue schizonts burst the infected hepatocytes releasing thousands of merozoites into the circulation. The tissue merozoites infect the erythrocytes, undergo a series of asexual multiplication cycles (erythrocytic stage), produce new infective merozoites which burst the erythrocytes and a new infective cycle begins. Some merozoites develop into male and female gametocytes. These are taken up when the next mosquito bites an infected person and mature in the mosquito gut. The gametocytes fuse to form an ookinete and the ookinetes develop into new sporozoites that migrate to the insect’s salivary glands, ready to infect the next vertebrate host. Reviewed in (12, 13).

#### Clinical Features

In high malaria transmission areas, many individuals, especially older children, and adults, carry asymptomatic parasitemia (14). Symptoms develop 7-10 days after the initial mosquito bite. Clinical disease manifests either as uncomplicated or complicated disease. Patients with uncomplicated malaria have fever, chills, headache, body ache, malaise, and vomiting. Severe or complicated malaria is a life-threatening disease. Patients have severe anemia, prostration, altered consciousness or coma, respiratory disease or metabolic acidosis, abnormal bleeding, hypoglycemia, repeated seizures, and acute kidney injury (12, 15).

Cerebral malaria is the most severe neurological complication of infection by *Plasmodium falciparum*. In children, coma develops rapidly with seizures following 1-3 days of fever. Status epilepticus is frequent and intracranial hypertension with brain swelling, retinal changes and abnormalities in posture and abnormal respiratory patterns are common. In some however, coma develops slowly with progressive weakness. Systemic complications include anemia, metabolic acidosis, electrolyte imbalance, hypoglycemia, and shock. Mortality is particularly high in patients with deep coma, severe metabolic acidosis, shock, hypoglycemia, and repeated seizures [reviewed in (16)].

On the other hand, cerebral malaria in adults is mostly part of a multi-organ disease. Patients progressively develop generalized weakness, delirium and coma and compared to the disease in African children, seizures, raised intracranial pressure and retinal changes are less common and coma resolution is slower.

### Pathophysiology of Cerebral Malaria

*a. How the parasites get to the CNS and the interplay between the parasites and the immune system in the CNS*

**Figure 1:**
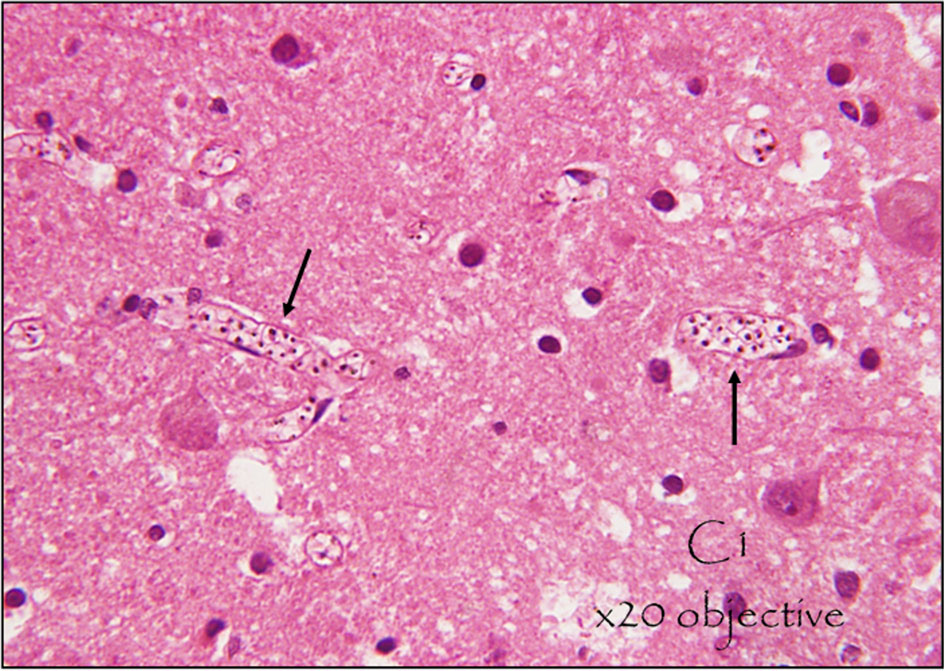
Sequestration of malaria parasites in cerebral micro vessels. This is a hematoxylin and eosin (H&E) stained section of the brain of a middle-aged male who died from cerebral malaria following 5 days of fever, vomiting, and difficulty in breathing, shock, and coma. Appreciate the parasites in the erythrocytes within the blood vessels (seen as black dots) at x20. Photo Courtesy of Dr. Robert Lukande, Makerere University.

The hallmark of cerebral malaria is intravascular sequestration of circulating parasitized erythrocytes in the cerebral microcirculation (17), **Figure 1**.

**Figure 2:**
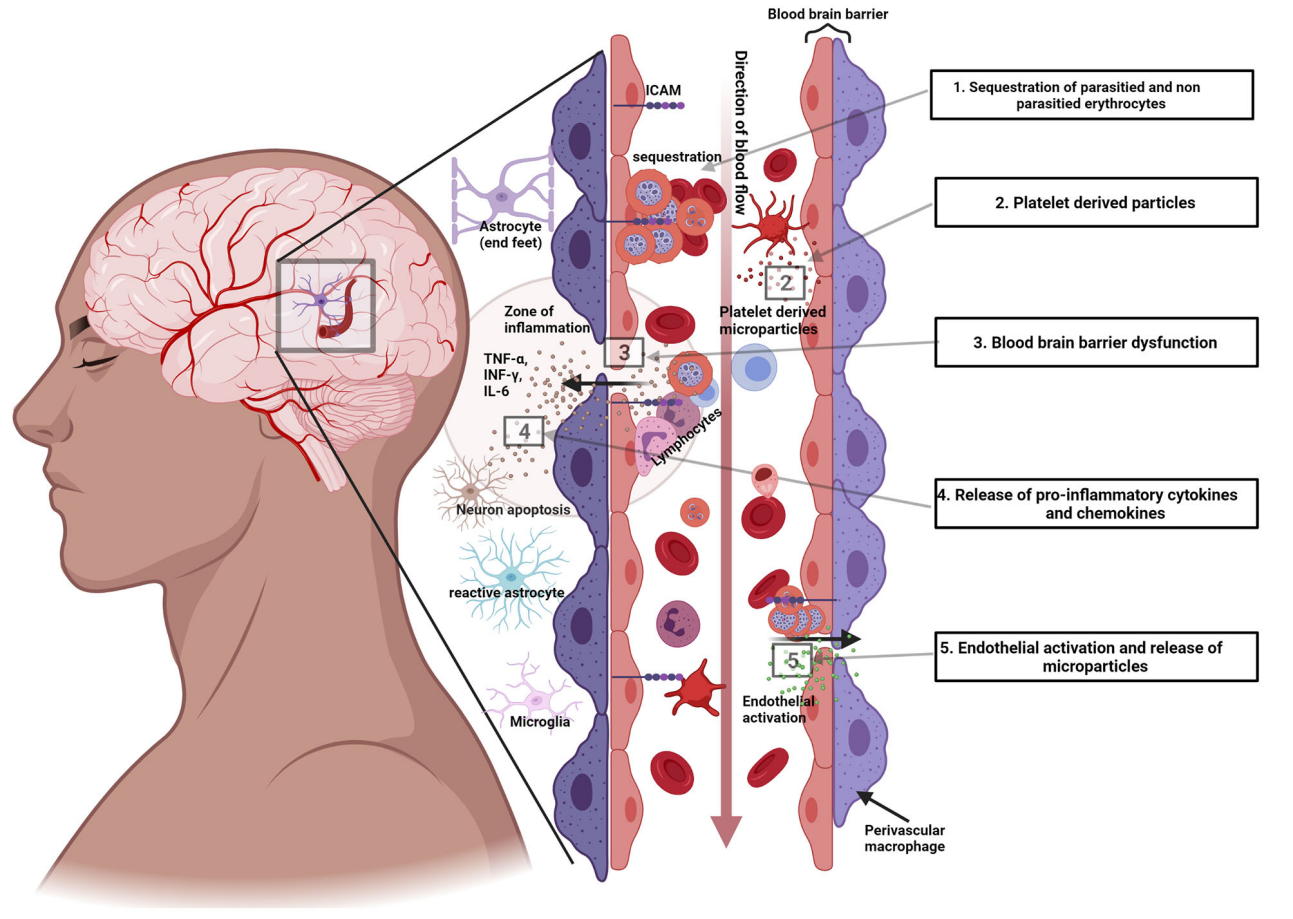
Illustration of some of the pathogenic mechanisms in cerebral malaria. 1) Sequestration of P. falciparum parasitized and non-parasitized erythrocytes. 2) Platelet derived microparticles released into the circulation. 3) Dysfunction of the blood brain barrier. 4) Release of proinflammatory cytokines and chemokines and cytokine and chemokine induced injury. 5) Endothelial activation.

Several processes, other than sequestration, are also implicated in the pathogenesis. These include microvascular obstruction by the sequestered erythrocytes, an excessive proinflammatory cytokine response, excitotoxic release, endothelial dysfunction, and dysregulation. The extent of the contribution of the specific mechanisms to disease remain to be elucidated, **Figure 2**.

Sequestration is due to cytoadherence of infected erythrocytes to the vascular endothelial cells *via* parasite derived proteins on the surfaces of the infected erythrocytes e.g., the *Plasmodium falciparum* erythrocyte membrane protein-1 (PfEMP1). These attach to ligands upregulated on the lining of the microcirculation. The sequestered mass is further increased when adherent cells bind other infected erythrocytes (autoagglutination) or non-infected erythrocytes (rosetting) or use platelets to bind other infected erythrocytes (platelet-mediated clumping). Encoded by up to 60 variant genes, PfEMP1 binds to several host receptors including CD36 and the intercellular adhesion molecule 1 (ICAM1) and binding of infected erythrocytes to ICAM1 is implicated in the pathogenesis of cerebral malaria. Indeed, postmortem studies have demonstrated the upregulation of ICAM1 expression on the cerebral vascular endothelium in cerebral malaria (18).

Sequestration reduces microvascular flow. The presence of parasites inside the erythrocytes further decreases erythrocyte deformability so that erythrocytes have increased difficulty in passing through the cerebral microvasculature (19). Hypoxia and reduced tissue perfusion have therefore been considered important pathophysiological mechanisms. However, significant neuron death is unlikely because with specific antimalarial treatment, coma, especially in children, is rapidly reversible. Despite this, in the presence of increased metabolic demand such as during seizures, the risk of neural injury is higher and may be worse if the patient is hypoglycemic or if blood flow is further compromised by intracranial hypertension [reviewed in (18)].

Cerebral vascular dysfunction is now considered a major process in the pathogenesis of cerebral malaria and a target for the development of adjuvant therapies. Even though the parasites remain largely intravascular, especially in children, they cause some disruption of the BBB function. There is a redistribution of tight junction proteins occludin, vinculin, and zonula occludens 1 (ZO-1), that are central to BBB integrity (20). On immunohistochemistry, BBB impairment is seen in areas of the infected erythrocytes, where they are associated with focal loss of endothelial intercellular junctions (21). Cerebrovascular endothelial cell activation, defined by increased ICAM1 staining and reduction in cell-junction staining, and disruption of junction proteins, particularly in vessels containing infected erythrocytes is observed (20) but such disruption has not been associated with significant leakage of plasma proteins into perivascular areas or in to the cerebrospinal fluid (21). Low levels of nitric oxide bioavailability, high levels of endothelin-1 and dysfunction of the angiopoietin-Tie2 axis are critical (22). In African children, the dysfunctional endothelial function is associated with brain swelling (23, 24) but increased cerebral volume is thought to be the main cause of intracranial hypertension (25).

Activation of the microvascular endothelium is associated with the release of endothelial microparticles. The concentration of microparticles in peripheral blood is a good correlate of the degree of endothelial activation in deep tissues (26). Most proteins associated with the microparticles in cerebral malaria are involved in localization processes and in response to stimuli, with the immune and inflammatory responses (27). The angiopoietins are important in maintaining the integrity of the endothelial lining. Dysregulation of angiopoietin-1 (ANG-1) plays a mechanistic role in the pathogenesis of cerebral malaria while plasma levels of especially ANG-2, positively correlates with disease severity. The ratio of ANG-2/ANG-1 predict fatal cerebral malaria (28).

In the brain, malarial parasites stimulate the production of proinflammatory cytokines which activate the endothelial cells prompting them to produce CXCL10, a chemoattractant for leukocytes. The accumulated platelets in the microvasculature also release CXCL4 from their alpha granules which in turn stimulate the production of tumor necrosis factor (TNF-α) by the local mononuclear leukocytes. Other proinflammatory cytokines, such as lymphotoxin-α (LTα), interferon-γ (IFN-γ), interleukin-1α (IL-1α), and IL-1β, are also upregulated [reviewed in (29)]. All these contribute to the heightened hyper-inflammatory state. *In vitro*, TNF-α induces the release of pro-coagulant and pro-adhesive microparticles from cultured endothelial cells and upregulates ICAM1 expression on endothelial surfaces which further induces the sequestration of parasitized erythrocytes in the cerebral microvasculature. However, although high plasma and CSF levels of TNF-α are associated with poorer outcomes, inhibition of TNF-α by anti-TNF antibodies did not improve outcome (30) but synthetic oleanane triterpenoids reduced plasma levels of IL-10, TNF-α, and IFN-γ (31), thereby enhancing the integrity of the brain blood barrier in experimental cerebral malaria.

Animal studies suggest that Toll-like receptors (TLR) may be involved in promoting cerebral malaria immunopathology; loss of TLR7 conferred partial protection against fatal disease, and loss of TLR signalling dysregulated the cytokine profile towards those with more anti-inflammatory properties (32). Genetics too, may play a role; alternative alleles in one gene may either favor or counteract the development of severe disease (e.g., HMOX1), and different genetic variants within a gene promoter are associated with different severe malaria syndromes (e.g., TNF) suggesting differential gene regulation in context of different inflammatory milieus [reviewed in (33)].

Recent findings suggest a role of CD8+ T cells (killer T cells) in BBB and BCSFB dysfunction (34, 35). In both mouse and human cerebral malaria, it has been noted that CD8+ T cells accumulate within brain vasculature particularly within the cortex compared to other regions (34, 36). Using mouse models particularly, the involvement of CD8+T cells in cerebral malaria pathology has been demonstrated. However, their exact role remains unclear (36). The prevailing hypothesis is CD8+ T cells interact with endothelial cells or epithelial cells *via* the T cell receptor and MHC class I, leading to tight junction disruption in both the BBB and BCSFB (35).

In addition, in both human and mouse cerebral malaria, oxidative stress (reactive oxygen species) is detected in the brain. Hemoglobin degradation by the malaria parasite produces the redox-active by-products, free heme, and H_2_O_2_, conferring oxidative insult (37, 38).

In summary, five sequential and complex inflammatory process to malarial infection contribute to the development of cerebral malaria. First, the two parasite replication phases, the hepatic and erythrocytic stages, lead to two distinct innate responses that modulate subsequent parasite and host cell interactions. These two are followed by sequestration induced endothelial activation and enhanced chemokine secretion, leukocyte recruitment and eventually permeabilization of the endothelial barrier (39), **Figure 2**.

*b. Consequences of this interplay between the parasites and the immune system*

The interplay between the parasites and the immune system is manifest in the severity of disease with severe metabolic derangement, deep coma, brain swelling and repeated seizures or status epilepticus. Parenteral artemisinins, artesunate in particular, is the first line specific treatment. Quinine is the alternative first line drug. A range of supportive treatments, including fluid therapy, glucose, blood transfusion, and anticonvulsants are needed to correct the deranged metabolic state and shock, correct anemia, and terminate status epilepticus. Despite treatment, death occurs in up to 20% of children and an even higher proportion of adults. In the long term, 25% of child survivors have long term neurological, cognitive and behavior disorders (16).

### Value of Neuroimmune Changes in Diagnostics and Therapeutics

In recent days, several investigative tools and biomarkers have become available for diagnosis and research, in helping to understand pathogenesis and examine emerging therapeutic approaches. The investigative imaging techniques include *in vivo* bioluminescent imaging, a versatile and sensitive tool that is based on the detection of light emission from cells or tissues, real-time *in vivo* imaging, F-fluorodeoxyglucose (FDG) positron emission tomography (PET) and intra-vital microscopy, a recently developed, advanced imaging tool that allows the direct and live visualization of the brain *via* a cranial opening [reviewed in (40)]. The technique can reveal cellular responses over time and space during the course of experimental cerebral malaria (41).

Several diagnostic tools have emerged around the concept of malaria retinopathy. Due to sequestration of the parasitized cells and the vascular changes associated with blood flow obstruction, the retinal microvasculature shows changes comparable to those occurring in the brain, making them an easily observable surrogate marker to assess pathology in cerebral malaria (42, 43). These include optical coherence tomography (an *in vivo* technique that allows optical-signal acquisition by which high-resolution cross-sectional images of the retina), optic nerve-head and the retinal nerve fiber layer are analyzed (44). Others are Teleophthalmology, Fluorescein retinal angiography and the micro-electroencephalogram.

As for biomarkers, three sets – a) screening and diagnostic markers that aid early diagnosis, b) prognostic biomarkers and c) those with potential for future research have emerged [reviewed in (40)].

The data on the coagulation-inflammation interface in cerebral malaria suggest these may be potential therapeutic targets for African children with cerebral malaria. They may include reducing thrombin generation with specific thrombin or prothrombinase antagonists, and augmenting the protein C pathway (45). Also, matrix metalloproteinases, a family of proteolytic enzymes involved in modulating inflammatory responses, disrupting tight junctions, and degrading sub-endothelial basal lamina, represent potential innovative drug targets (46). However, the multifaceted pathogenic mechanisms and absence of therapeutics against the inflammatory responses to date still account for the failure to reduce morbidity and mortality.

### Knowledge Gaps

A lot remains to be learnt on the pathogenesis of cerebral malaria. It should be noted that although mice models have been used to study the pathogenesis, the pathology in mice is different; infected erythrocytes do not commonly sequester; instead, monocytes occur in cerebral vessels, and inflammatory cytokines are essential for the pathogenesis.

Despite emerging information about specific parasite subtypes, the intravascular processes leading to cerebral malaria remain to be determined. It has been the understanding that the malaria parasite was not able to penetrate actual brain tissue, but emerging information suggest that malaria parasites can do just that and a recent study mapped the mechanisms they utilize (47). This discovery points to parasites in the brain endothelium as a contributing factor to the pathology of human cerebral malaria. This new line of study urgently needs to be expanded.

The role of both the parasite and host genetics in the development and presentation of disease is poorly understood. For example, in mice models, absence of ApoE, a dominant apolipoprotein in the brain that has been implicated in several neurological disorders, was associated with decreased sequestration of parasites and T cells in the brain (48). Do similar alleles play such roles in humans?

Lastly, other than specific treatment, to date, most adjuvant intervention studies have been disappointing. New approaches are urgently required.

## Neuroimmunology of Human African Trypanosomiasis

### Introduction

#### Pathogen Description

Human African trypanosomiasis (HAT), also known as sleeping sickness, is a disease endemic to Sub Saharan Africa (SSA) caused by two subspecies of a microscopic flagellate protozoan parasite *Trypanosoma brucei (T. b.)*, which are *T. b. gambiense* and *T. b. rhodesiense*. *Trypanosoma brucei* is a unicellular extracellular parasite found in blood or other body fluids of the host such as the lymph and cerebrospinal fluid (CSF) (49). The parasites are transmitted by infected tsetse flies (*Glossina* sp.*)* while feeding on blood. These tsetse flies are found only in the SSA region; thus, transmission can only occur in this region. *T. b. gambiense*, which currently accounts for 98% of HAT (50), is found in large areas of central and western Africa and is considered an anthroponotic disease (51). On the other hand, *T. b. rhodesiense*, which accounts for about 2% of the disease, has limited distribution in eastern and southern Africa is a zoonotic disease, infecting mainly wild animals and livestock (50, 51). Another subspecies *T. b. brucei* is not human pathogenic and thus used extensively in research using animal models of HAT. The three subspecies are morphologically indistinguishable.

#### Signs and Symptoms

HAT is divided into two clinical stages: an early hemolymphatic phase, also referred as stage 1, and a late meningo-encephalitic phase, also referred to as stage 2. In stage 1 some patients develop a chancre at the bite site of inoculation of the parasite followed by involvement of blood and lymphatic systems with general symptoms of infection including chronic intermittent fever, headache, asthenia, lymphadenopathy, and pruritus. In stage 2 patients have more neurological symptoms such as sleep disorders (described in more detail in the article by Ngarka et al. in this collection), confusion, tremor, general motor disturbances, sensory disturbances, abnormal movements, and speech disorders as well as psychiatric symptoms (52–54). *T. b. gambiense* HAT is more chronic lasting months to several years between infection and death, whereas *T. b. rhodesiense* HAT is more acute lasting several weeks to months, such that in the latter the demarcation between the early and late stages of the disease are less clear (51, 55–57). If untreated the disease leads, in most patients, to cachexia, opportunistic infections, coma and eventually death (53).

#### Diagnosis

Clinical presentation is non-specific, thus, diagnosis of HAT is confirmed by finding trypomastigotes in blood, lymph (early stage) or CSF (late stage) using microscopy. Serological tests (card agglutination test for trypanosomiasis, CATT) are available for screening for *T. b. gambiense*, whereas there are no serological tests for *T. b. rhodesiense*. The WHO criteria for CNS involvement include the presence of CNS symptoms and finding parasites in the CSF or a WBC count of >5/μl (53, 58). However, some countries use a CSF WBC count of > 20/μl (59, 60). Thus, there is a grey zone where it is not clear what finding WBC counts of >5 & <20/μl mean (54, 61). This has led to a search of biomarkers to better stage the disease, some of which are discussed below.

#### Treatment

The drugs used for the treatment of HAT have for a long time been divided into drugs for early stage, suramin for *T. b. rhodesiense* and pentamidine for *T. b. gambiense*, and drugs for late stage, melarsoprol for *T. b. rhodesiense*, eflornithine and the nifurtimox-eflornithine combination (NECT) treatment for *T. b. gambiense* (57). These are all administered intravenously except for nifurtimox, which is given orally as part of NECT. However, a new orally administered drug fexinidazole, was recently introduced to treat both early and late stages of *T. b. gambiense* HAT (50, 62, 63). Melarsoprol and NECT penetrate the BBB better and thus they are more effective, however, they are more toxic and have more complex dose regimens than suramin and pentamidine. Acoziborole, another drug administered orally as a single dose is under clinical trials for *T. b. gambiense* HAT with promising results (62).

#### Prevalence

The number of incident cases of HAT fell to below 1000 in 2018; thus, the WHO aim of elimination of HAT i.e., less than 2,000 reported *T. b. gambiense* HAT cases by 2020 has been met (50, 63, 64). This has been because of a concerted effort on surveillance, medical treatment, and interruption of transmission by the WHO, local governments, many NGOs and public-private partnerships such as that with Sanofi-Aventis and Bayer, the latter donated the necessary drugs to treat HAT (63, 64). However, still there are about 70 million people at some risk of HAT in SSA countries (65). There is a need for continuous surveillance and control programs because there can be a resurgence of HAT. HAT was well controlled during the 1960s but when surveillance was reduced because of disturbances due to wars as well as reduced resources to control HAT the cases went up reaching a peak in the 1990s till interventions were brought about to control it (51, 57, 63, 66).

### *Trypanosoma brucei* spp., Immune System and Neuropathogenesis

Several recent reviews have given a more extensive description on the neuropathogenesis of HAT (54, 67–69). This section will focus on the interplay between the parasites and the immune system in the CNS. Stage 2 of HAT is characterized by CNS involvement in the symptomatology of the disease and neuroinflammation. Trypanosomes have been difficult to find in *post-mortem* studies of brains of HAT patients, possibly because of autolysis, lack of proper antibodies for staining the parasites, or clearance due to drug treatment (54, 70, 71). Trypanosomes were observed in the brain parenchyma during autopsy of a *T. b. rhodesiense* HAT patient who had an acute disease and died before treatment (72). Neuroinflammation is a characteristic feature observed in the brain during *post-mortem* of HAT patients. Perivascular and white matter infiltration by inflammatory cells, predominantly mononuclear lymphocytes, has been described (54, 68, 70, 73). Morular-shaped plasma cells loaded with immunoglobulins (Mott’s cells) are also found in the brain (54, 68). The leukoencephalitis caused by infiltrating cells is also accompanied by microglia and astrocyte activation (68). There are also changes in the monoaminergic neurotransmitters, dopamine, serotonin, and norepinephrine, in the brain during trypanosomiasis, which might contribute to the neuropsychiatric abnormalities observed in HAT (74, 75).

#### How the Parasites Get to the CNS

Information about how the parasites enter the brain and neuroimmunological changes that occur has been obtained from experiments done principally with rodent models of HAT (54, 66, 68). In rats and mice models of HAT infected with *T. b. brucei*, parasites invade the choroid plexus and circumventricular organs such as the area postrema, pineal gland, and median eminence (76), that lack a BBB, at early stages of the infection. At later stages post-infection, parasites penetrate the BBB and invade the brain parenchyma mainly in the white matter and the septal nuclei than the cerebral cortex, while the tight junction proteins are preserved (77). Double immunohistochemical labeling of parasites and brain endothelial cells (using antibodies against glucose transporter-1 (GLUT-1) in the brains of mice or rats infected with *T. b. brucei* was used to visualize the location of parasites, either inside blood vessels or in the brain parenchyma (77–81).

A study utilizing two different mice strains, C57BL/6 and SV-129/Ev mice, showed that host genetic differences in the expression of immune molecules determine parasite invasion of the CNS (80). C57BL/6 mice infected with *T. b. brucei* had less parasitemia but more T cells and parasites in the brain parenchyma than SV-129/Ev mice. The C57BL/6 mice also had higher IgM in the serum and higher proinflammatory cytokines and adhesion molecules in the brain than SV-129/Ev mice (80).

A series of studies using immunodeficient mice or mice deficient of various cytokines, chemokines, other inflammatory molecules and their signaling molecules elucidated the role of the immune system in the passage of the parasites and T cells across the BBB into the brain parenchyma [see **Table 1** and described in detail in (54)]. In summary, during infection immune cells are activated in a TLR-MyD88 dependent manner and produce cytokines such as TNF-α and IFN-α/β, which are important for control of parasitemia but also possibly for initiation of T cell and parasite invasion of the brain, and for control of parasites in the brain parenchyma (54, 79). TNF-α induces the expression of adhesion molecules, while IFN-α/β-induces limited expression of C-X-C motif chemokine ligand 10 (CXCL10), which facilitates T cell and parasite invasion of the CNS (78, 79). The parasites require T cells and IFN-γ to cross the BBB (81). IFN-γ induces CXCL10 which attracts and/or retains T cells and the parasites in the brain parenchyma (78). IFN-γ possibly induces matrix metalloproteinase-9 (MMP-9) to facilitate T cells and parasites crossing of the parenchymal basement membrane (82). On the other hand, nitric oxide (NO) produced by inducible nitric oxide synthase (iNOS) is important for maintaining the integrity of the BBB and prevent unlimited T cell and parasite invasion of the brain (82).

**Table 1:** Immune cells, molecules and their signaling molecules involved in *Trypanosoma brucei brucei* neuroinvasion.

Immune cells or molecules	Immune cells and trypanosome levels in the brain parenchyma of transgenic mice compared to WT mice	Proposed role	Ref.
**Immune cells**			
B and T cells	*Rag1* ^–/–^ mice, which lack mature T and B cells, had less trypanosomes in the brain parenchyma compared with WT mice. Trypanosomes accumulated in the perivascular compartment, confined between the endothelial and the parenchymal basement membranes, in certain areas of the brains of the transgenic mice	Facilitate parasite crossing of the BBB into the brain parenchyma. Necessary to produce IFN-γ during infection.	(81)
**Chemokines and their receptors**			
CXCL10	*Cxcl10* ^-/-^ and *Cxcr3* ^-/-^ mice had less T cells and trypanosomes in the brain parenchyma compared with WT mice.	Chemoattraction, recruitment or retention of T cells and trypanosomes in the brain parenchyma	(78)
**Cytokines and their receptors**			
IFNα/β	*Ifn-α/βr* ^-/-^ mice had less T cells and slightly less trypanosomes in the brain parenchyma compared with WT mice.	Facilitate sensitized T cells and a few parasites crossing of the BBB (more of initiation of the process) by inducing a limited release of CXCL10 from astrocytes and endothelial cells	(79)
IFN-γ	*Ifn-γ* ^-/-^ and *Ifn-γr* ^-/-^ had less T cells and trypanosomes in the brain parenchyma compared with WT mice. Trypanosomes accumulated in the perivascular compartment, confined between the endothelial and the parenchymal basement membranes, in certain areas of the brains of both transgenic mice.	Facilitate T cell and parasite crossing of the BBB in part by inducing the expression of CXCL10. Other mechanisms remain to be elucidated	(81)
IL-12	*Il-12p40* ^-/-^ mice had less T cells and trypanosomes in the brain parenchyma compared with WT mice. There was sporadic clustering of trypanosomes around vessels.	Facilitate T cell and parasite crossing of the BBB by inducing the expression of IFN-γ	(81)
TNF-α	*Tnfr1^−/−^* mice had less T cells and trypanosomes in the brain parenchyma compared with WT mice.	Facilitate T cell and parasite crossing of the BBB by increasing expression of adhesion molecules i.e., ICAM-1	(79)
**Toll-like receptors**			
TLR2 and TLR9	*Tlr2-/-* mice had similar T cells but more trypanosomes in the brain parenchyma of the corpus callosum compared with WT mice. *Tlr9-/-* mice had less T cells in the brain parenchyma compared with WT mice. However, they had more trypanosomes in the brain parenchyma of the corpus callosum and less in the septum. *Tlr2/9-/-* mice had less T cells but more trypanosomes in the brain parenchyma compared with WT mice	T cells cross the BBB after they are activated in secondary lymphoid organs in a TLR-dependent manner and might pave way for trypanosomes. However, TLR dependent signaling is essential for parasite control in the brain	(79)
**Intracellular signaling mediators**			
MyD88	*Myd88*-/- had less T cells but more trypanosomes in the brain parenchyma compared with WT mice	T cells cross the BBB after they are activated in secondary lymphoid organs in a MyD88-dependent manner and might pave way for trypanosomes. However, MYD88 dependent signaling is essential for parasite control in the brain	(79)
**Nitric oxide**			
iNOS	*Inos*-/- mice had more T cells and trypanosomes in the brain parenchyma compared with WT mice	iNOS-generated NO by perivascular macrophages prevents unlimited invasion of the brain parenchyma by T cells and parasite by maintaining the integrity of the BBB	(82)

BBB, blood-brain barrier; CXCL, C-X-C motif chemokine ligand; IFN, Interferon; IL, Interleukin; iNOS, inducible nitric oxide synthase; MyD88, Myeloid differentiation primary response 88; TLR, Toll-like receptor; TNF, Tumor necrosis factor; RAG-1, recombinant activating gene 1; WT, Wild type. Adapted from (83) and (84).

#### Neuroinflammation: Interplay Between the Parasites and the Immune System in the CNS

**Figure 3:**
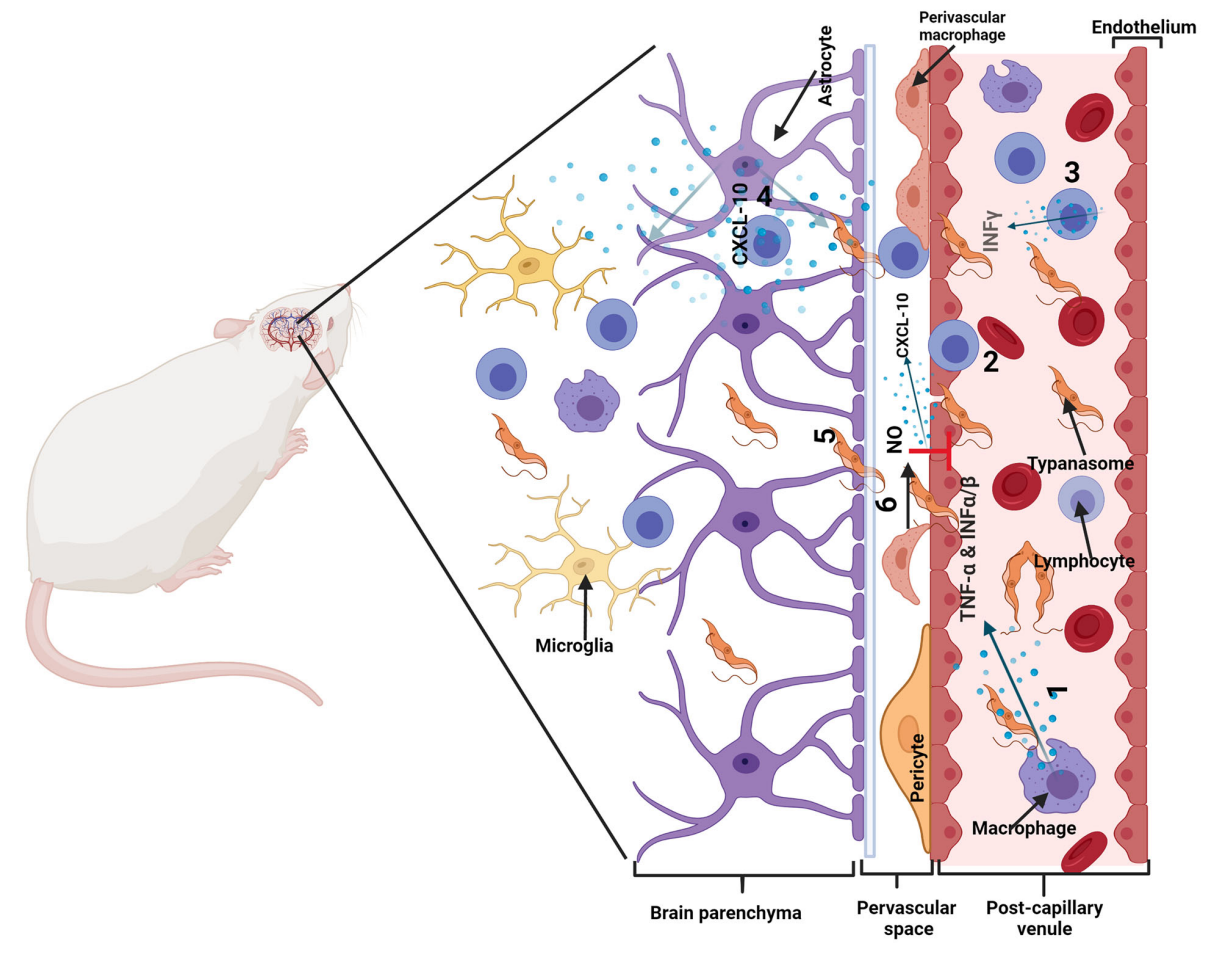
Illustration of neuro-pathogenic mechanisms of trypanosomes: Trypanosoma brucei spp. and T cells cross the blood-brain barrier (BBB), in a multistep manner to invade the brain parenchyma. In infected mice, activated immune cells and astrocytes release molecules that cause T cell and trypanosomes to cross the barriers of endothelial cells linked by tight junctions, the endothelial basement membrane (BM) and the parenchymal BM into the brain parenchyma. 1. Immune cells are activated in a TLR-MyD88 dependent manner and produce cytokines such as TNF-α and IFN-α/β. 2. TNF-α induces the expression of adhesion molecules such as ICAM/VCAM on cerebral endothelial cells which allow attachment of T cells. IFN-α/β induces a limited release of CXCL10 by endothelial cells and/or astrocytes, which is enough for penetration of T cells accompanied by some trypanosomes into the perivascular space. 3. Trypanosome-derived antigens taken up and expressed by macrophages are recognized by sensitized T cells to induce IFN-γ, which amplifies the process through 4. induction of CXCL10 production by astrocytes and 5. molecules that open the parenchymal basement membrane for spread of both T cells and trypanosomes into the brain parenchyma (In the absence of IFN-γ-inducible molecules, the parasites and T cells do not penetrate the parenchymal BM efficiently and they accumulate within the perivascular space as cuffs). 6. Nitric oxide (NO) generated in perivascular macrophages by iNOS can inhibit the effects of TNF-α and prevent unlimited immune cell and parasite invasion of the brain parenchyma. Modified from (54) with permission from Elsevier to reuse.

Invasion of the CNS is dependent on T cells and accompanied by T cell infiltration of the parenchyma (81). This elicits an inflammatory response in the brain with activation of microglia and astrocytes, which produce cytokine, chemokines, and NO (78, 82, 85–89). Activated astrocytes increase the expression of the chemokine CXCL10, which is important for the recruitment and retention of T cells and parasites (79). There is an increased production of other chemokines such as chemokine (C-C motif) ligand 2 (CCL2), CCL5, CXCL9, CXCL13 (78, 90, 91). There is also a robust upregulation of inflammatory cytokines such as IL-1β, IL-6, IFN-γ, TNF-α (81, 86, 89, 90),. Other inflammatory molecules such as iNOS are also upregulated (82, 92, 93). Although, the chronic inflammation is detrimental to the brain it is also important for suppressing parasite numbers and maintaining the integrity of the BBB. TLR-MyD88 dependent signaling is important for parasite control (79) and NO derived from iNOS is important for maintaining BBB integrity and limiting the invasion of the brain parenchyma by parasites and T cells (82). Some cytokines such as IL-6 and IL-10 have also been shown to reduce systemic IFN-γ and TNF-α, reduce number of trypanosomes in the CNS and to protect against the neuroinflammatory pathology that occur during infection (94) (**Figure 3**).

#### Consequences of This Interplay Between the Parasites and the Immune System

Microglia activation is concomitant with onset of sleep disorders in mice (85). Microglia and astrocytes together with lymphocytes could cause disturbances through increased expression of cytokines. Cytokines such as IFN-γ and TNF-α have been proposed to contribute to some of the neurological disturbances observed in HAT such as hyperalgesia, sleep disturbances and alteration in circadian rhythm, covered in detail in previous reviews (54, 66).

### Value of Neuroimmune Changes in Diagnostics and Therapeutics

There has been great interest in evaluating the cytokine and chemokines upregulated in the CNS during HAT and in animal models as biomarkers for disease staging and monitoring therapeutic outcomes (54, 68, 95). CXCL10 was considered as a candidate marker for late-stage HAT (78), and the sensitivity was increased by combining it with H-FABP and CXCL8 (96), CXCL13 and MMP-9, or CXCL13 and IgM (97). Other immune and inflammation related molecules that have also been evaluated as biomarkers for staging HAT include IgM (98, 99), IL-10 (98–100), CXCL13 (101, 102), MMP9 and ICAM-1 (103).

The new oral drug, fexinidazole, is now available for the treatment of *T. b. gambiense* HAT. However, the treatment of second stage *T. b. rhodesiense* HAT is still reliant on the arsenic compound melarsoprol, which is highly toxic, producing post-treatment reactive encephalopathy (PTRE) in about 10% of the patients, which can be fatal in up to 50% of these cases (104, 105). PTRE exacerbates the neuroinflammation that already exists in HAT such as astrogliosis and the increased presence of immune cells such as lymphocytes, macrophages, and plasma cells in the brain white matter (70, 104). Minocycline was found to prevent *T. b. brucei*-induced microglia and astrocyte activation as well as the expression of inflammatory molecules in the brain (87). Immunomodulators such as minocycline warrant to be evaluated for the prevention of PTRE when given in combination with melarsoprol.

### Knowledge Gaps

An intermediate stage of HAT, between stage 1 and stage 2 has been suggested (106) and a recent study using CXCL13 as one of the biomarkers supports its existence (101). More studies are needed to characterize this stage, which sometimes respond to stage 1 drugs, in terms of presence or absence of parasites in the brain parenchyma, other possible biomarkers and appropriate treatment regimens.

IFN-γ-induced CXCL10, which is important for T cells and parasites chemoattraction and retention in the brain parenchyma (78), has come out as a strong candidate biomarker for staging HAT. IFN-γ induces other molecules, to facilitate T cells and parasite crossing of the BBB, whose nature is yet to be determined. Finding these molecules induced by IFN-γ could be important both from the pathophysiological point but also to find possible biomarkers for staging HAT.

In conclusion, the immune system plays a role in the role in the neuropathogenesis of HAT and side effects of melarsoprol. Immune related molecules such as the chemokines CXCL10 and CXCL13 are coming out as useful biomarkers for staging HAT. Targeting neuroinflammation with immunomodulators such as minocycline warrant further studies to reduce the incidence and mortality of melarsoprol-induced PTRE.

## Cerebral Toxoplasmosis in Africa

### General Features of *T. gondii* and Toxoplasmosis

#### Pathogen Description

The single-celled Apicomplexan *Toxoplasma gondii* has felines (cats) as it’s definitive hosts (107). However, it was first described in the North African rodent *Ctenodactylus gundi* (108). In humans, ingested oocyst stages (originating from cat feces) or tissue cysts (from meat containing the bradyzoite stage) invade the intestinal epithelium and transform into rapidly replicating tachyzoites. Tachyzoites are obligate intracellular for replication and disseminate rapidly in the organism before differentiating back into bradyzoites, which form persistent intracellular cysts. As chronic infection sets in, tissue cysts form preferentially in the brain and retina of intermediate hosts, for example in humans, rodents and in animals used for meat consumption. At the chronic stage of infection, the immune system plays a critical role in controlling parasite loads but can also contribute to detrimental inflammation (109).

#### Prevalence of Carriage of *T. gondii* in Africa

Parasite population structure studies have shown that a few major clonal lineages of *T. gondii* predominate worldwide (110). Consistent with this, lineages shared with other global geographical areas are present in Africa and unique African haplogroups (111–113). Studies of seroprevalence indicate that carriage of *T. gondii* is common in the African population (114, 115), with recent reports indicating that seroprevalences vary broadly depending on region and age and range on average between 30-70% (116–121). The seroprevalence levels in humans go together with the reported prevalence in animals used for meat consumption (113, 122).

#### Clinical Symptoms and Manifestations

In healthy individuals, primary *T. gondii* infection is normally asymptomatic or accompanied by mild flu-like symptomatology, such as fever, malaise and swollen lymph nodes (123). In contrast, upon primary infection in pregnant women, the parasite can transmit across the placenta and cause neurological damage or even be fatal to the developing fetus (124). In conditions associated with immunodeficiency, such as HIV/AIDS or immune-suppressive therapies, reactivation of tissue cysts in the brain can cause life-threatening toxoplasmic encephalitis (TE). In contrast, ocular toxoplasmosis (OT) can manifest as retinochoroiditis in otherwise healthy immune-competent individuals. Following congenital transmission, OT can relapse repeatedly many years later in life (124).

### Pathophysiology of Toxoplasmosis

#### Invasion to the Brain Parenchyma

Strictly, how *T. gondii* enters the human brain is largely unknown and the current paradigms are therefore based on extrapolations from infections in rodents (109). Overall, *T. gondii* infection has a clear predilection for the CNS, including congenital infection, which is likely linked to the fact that cysts chronically persist in the CNS while they are cleared from peripheral organs over time. Thus, *T. gondii* consistently and silently manages to establish chronic infection in the CNS.

To date, molecular mechanisms that define tropism of *T. gondii* for the CNS over other organs have not been identified. Precisely how *T. gondii* gains access to neurons in the brain parenchyma and the mechanisms for chronic persistence within neurons remain enigmatic (125). Following invasion across the intestinal wall and systemic dissemination in the blood, *T. gondii* likely crosses the parenchymal vasculature of the blood-brain barrier (BBB) by different routes (126). A recent study in mice showed that passage occurs preferentially across cortical capillaries, while invasion across post-capillary venules, arterioles, the choroid plexus and meningeal vessels is less frequent (127). Paracellular entry across endothelium implies passing cellular tight junctions (128–131) while transcellular entry may occur after apical parasite invasion of the endothelium and basolateral exit after replication (132–134). Additionally, infected leukocytes, such as parasitized dendritic cells, may mediate transportation into the parenchyma (135–137).

#### Interplay Between the Parasite and the Immune System in the CNS

The crucial roles that immune surveillance plays in the manifestations of toxoplasmosis is best illustrated by the fact that AIDS patients with low CD4^+^ T cell count (< 200/μl) and seropositive for *T. gondii* are at risk of developing a reactivated TE due to the loss of T cell-mediated control of brain cysts (124). Similarly, individuals receiving immunosuppressive treatments, for example after organ transplants, are at risk of developing a reactivated toxoplasmosis. Thus, immune-mediated control is closely linked to the pathogenesis for this opportunistic infection.

##### Control of the Infection by Immune Responses and Immunopathology

Overall, the systemic response to *T. gondii* is characterized by a strong Th1-type immune response that is dominated by production of proinflammatory mediators such as IL-12, IFN-γ, TNF and NO.

As the parasite colonizes the CNS, inflammatory leukocytes are recruited (138). In rodent infections, the inflammatory infiltration is constituted by CD4^+^/CD8^+^ T cells, Ly6C^high^ inflammatory monocytes, F4/80^+^ macrophages and CD11c^+^ DCs. T cells have a protective effect by secretion of cytokines, principally IFN-γ and TNF. In contrast to responses in mice (8), the initial steps for innate immune sensing in humans remain uncharacterized. However, a role has been recently identified for alarmin S100A11 which is released by *T. gondii*-infected cells and sensed by human monocytes (139). Further, IFN-γ-induced 2, 3 indoleamine dioxygenase (IDO) contributes to parasite control by human astrocytes (140). By counteracting the effects of proinflammatory cytokines, immune-suppressive cytokines likely also play a role in dampening immunopathology. In rodent neuro-toxoplasmosis, monocytic cells, microglia and B/T cells produce IL-10 (141–143). In acute and reactivated human OT, a disbalance between regulatory and proinflammatory T cell populations may account for the immunopathology (144).

##### Control of Infection by Brain Parenchymal Cells

Knowledge on immune mechanisms leading to the control of intracerebral *T. gondii* in humans is mainly based on extrapolations from reactivated toxoplasmosis in AIDS patients.

In addition to an abundant leukocyte infiltration in the brain parenchyma, astrogliosis and microglial nodules are not unusual findings upon human cerebral toxoplasmosis (145–147). This is indicative of the implication of astrocyte and microglia responses in human TE.

Important roles in the control of TE have been attributed to astrocytes in rodents. These include pro- and anti-inflammatory responses to balance parasite control and intracerebral immune responses to limit neuroinflammation and prevent neuronal damage (148–152). Similarly, microglia exhibit activation by secreting cytokines upregulating MHC class I/II molecules (153–155). Microglia can also suppress the proliferation of parenchymal T cells, and thus may contribute to reducing immunopathology (156).

Finally, the roles of neurons, which are the cells primarily targeted by *T. gondii* and that harbor the tissue cysts (157), remain unresolved in humans. *In vitro*, neurons respond with cytokine secretion (IL-6, TGF-β_1_, CCL3 and CCL4) upon *T. gondii* challenge (156), however, their role in parasite control remains unclear *in vivo*. In mice, it was suggested that parasite cyst-harboring neurons may escape perforin-dependent elimination by CD8^+^ T cells because neurons may remain MHC class I negative (158). More recent work showed that neuronal MHC I presentation was required for robust control of *T. gondii* in the CNS (159).

It has been proposed that an interplay between neuroinflammation and neurotransmission may underlie cognitive changes associated with chronic toxoplasmosis (160). Indeed, reported neurotransmission alterations during toxoplasmosis in rodents include dysregulations of catecholamines, GABA and glutamate (161–163).

These responses may implicate both neuronal and non-neuronal cells and await further investigations in humans.

#### Manifestations of Neurological Disease

While primary infection with *T. gondii* is asymptomatic or followed by mild symptoms, reactivation of chronically carried parasites is generally accompanied by neurological and ocular manifestations (123). In mice, Toxoplasma cysts can be sporadically localized in any anatomical cerebral area (164) and it may be assumed this is also the case for humans. Consequently, the clinical neurological manifestations will depend on the anatomical localization of the area of reactivation and on the parasite spread within the CNS. Individuals with severe immunosuppression are at a risk of developing TE and the encephalitic clinical presentation can range from lethargy to coma, incoordination to hemiparesis, memory loss to severe dementia, and focal motor to generalized seizures (165). Main risk groups include individuals with AIDS, but also individuals with organ transplants (166).

TE ranks among the most common neurological infections associated with AIDS. It has been estimated worldwide that 1/3 of AIDS patients seropositive for *T. gondii* and with low T cell count (< 200/μl) develop reactivated TE (124). While data is limited, African studies indicate that TE remains a major problem associated to AIDS, with variability among regions and countries (167–170). The strong association of TE with HIV/AIDS likely depends on failure to control CNS-resident parasites due to compromised antiparasitic T cell responses. Bradyzoite to tachyzoite conversion is accompanied by fast intracellular parasite replication that can result in necrotizing TE. Of note, *T. gondii* persists intracellularly in neurons, which can be MHC negative, and therefore likely not directly targeted by T cells in this respect.

Importantly, primary infection during pregnancy puts the developing fetus at risk of diverse neurological and ocular manifestations due to its immature immune system. Early infection during pregnancy can cause more severe neurological damage in the fetus and eventually abortion, whereas late infections generally cause less severe symptoms (171, 172). Fetal immune responses are largely uncharacterized, while transferred maternal *T. gondii*-specific antibodies likely contribute to protection.

### Value of Neuroimmune Changes in Diagnostics and Therapies

Blood serologic tests are broadly used in Africa for general diagnostics (116–120). Further, detection of *T. gondii* DNA by PCR in the cerebrospinal fluid is of high diagnostic value for CNS manifestations (173). Although not broadly applied, radiological methods, for example computer tomography, can provide differential diagnosis with other CNS conditions such as lymphoma, mycobacterial and fungal infections (cryptococcosis). Typically, single, or multiple rim-enhancing lesions with oedema, often in basal ganglia and white and grey matter zones are observed (174). Histopathological examination demonstrating tachyzoites of *T. gondii* or tissue cysts is also of value.

### Knowledge Gaps

#### Diagnostics and Risk Evaluation

The association between the different parasite genotypes and disease manifestation, especially cerebral or ocular disease and congenital transmission, remains unresolved (175). Given the contextuality of the clinical spectrum, the role of human genotypes needs also to be explored, especially in relation to immune surveillance and reactivation. Jointly, the identification of genetic risk factors for reactivated TE in AIDS or for congenital transmission could benefit risk groups and provide health care with tools for risk evaluation (173).

#### Therapies

Available treatments eliminate acute stage parasites (tachyzoites) (176). To date, drug resistance is not a considerable problem for *T. gondii* infection. Instead, a major problem is that chronic tissue cysts in the CNS are not eliminated by currently existing drug treatments. Therefore, a major advance would be the identification of druggable targets for the bradyzoite cysts (177) because it could potentially eliminate the severe and potentially lethal manifestations of reactivated disease in the CNS. Further, carriage of *T. gondii* has been linked to diverse neuropsychiatric conditions, for example schizophrenia (178), and antiparasitic therapies eliminating cysts may benefit these carriers.

#### Parasite Control and Vaccines

Strategies aiming at disrupting the parasite´s life cycle by preventing oocyst formation in felines or prevention of tissue cyst formation in intermediate hosts used for meat consumption would be of major benefit. To this end, a further understanding of the life cycle in felines and of immunity in intermediate hosts, including humans, is needed (179).

## Neurocysticercosis in Africa

### General Features of *T. solium* and Neurocysticercosis

#### Pathogen Description

*Taenia solium* is more commonly known as the pig tapeworm. It is a cestode belonging to the class Cestoda along with other flat, segmented, ribbon-shape worms. The larval stage of *T. solium* are fluid filled cysts with an invaginated scolex known as cysticerci, which typically infect pigs, the intermediate hosts of the parasite. The adult worm of *T. solium* is found in the small intestine of humans, the only known definitive host of the parasite. These worms can produce tens of thousands of oncospheres (eggs) per day, which are then excreted in feces where they contaminate food and water supplies. In the typical lifecycle, these oncospheres are ingested by pigs, where they are activated by digestive enzymes and bile salts. They then migrate through the intestinal wall into the blood supply. At blood vessel terminations within multiple different tissue types (e.g. muscle, subcutaneous or nervous tissue) they develop into vesicular larvae over the course of weeks to months (180). If insufficiently cooked pork meat containing a cysticercus is then ingested by a human, the scolex evaginates in the small intestine and attaches to the intestinal wall where it becomes an adult worm.

Infection of the human nervous system occurs when a human becomes an accidental intermediate host by ingesting oncospheres in contaminated food or water, often due to an adult tapeworm carrier in the household. The oncospheres are then activated in the human digestive tract just as they are in the pig, enabling them to pass into the blood stream and lodge in various tissue types including muscle, subcutaneous tissue, eyes and particularly the central nervous system. When cysticerci are present in the nervous system this is referred to as neurocysticercosis (NCC).

#### Prevalence of *T. solium* in Africa

*T. solium* is endemic to almost all sub-Saharan African countries with reports of *T. solium* taeniasis or cysticercosis having been made in at least 29 countries in Africa (181). Prevalence is thought to be minimal or non-existent in North African countries due to a combination of a dry climate, which doesn’t favor pig rearing, together with religious and cultural practices which preclude the consumption of pork. The presence of *T. solium* in a region can be ascertained by observing pigs infected with cysticerci (porcine cysticercosis), humans infected with adult tapeworm (taeniasis), or humans infected with cysticerci (cysticercosis and neurocysticercosis). The latter are often hard to diagnose as adults with taeniasis and cysticercosis are typically asymptomatic, whilst neurocysticercosis requires expensive, largely unavailable neuroimaging (e.g., CT scans) for definitive diagnosis (182, 183). As a result, the condition is typically underdiagnosed. Nonetheless, there are regions where *T. solium* is hyperendemic. For example, in the Eastern Cape region of South Africa, approximately 55% of pigs have cysticercosis (184) and up to 10% of people may have taeniasis or cysticercosis (185, 186).

#### Clinical Symptoms and Manifestations

Seizures are the most common symptom of NCC accounting for between 70 and 90% of all symptomatic NCC cases (187). Other symptoms include headaches, intracranial hypertension, hydrocephalus and meningitis (188). As an indication of the prevalence of NCC in endemic areas, approximately 29% of people with epilepsy have NCC (183). It is estimated that between 20 and 50% of all adults, acquired epilepsy in endemic countries is due to NCC (183, 189, 190). As a result NCC is one of the leading causes of adult-acquired epilepsy globally (191) and one of the most common neurological disorders in Africa (183, 189). Interestingly, in people with NCC, seizures often take months to years to develop following infection. This has led to the intriguing observation that while larvae are alive and viable within the brain, infected individuals are typically asymptomatic (192).

### Pathophysiology of Neurocysticercosis

#### Invasion to the Brain Parenchyma

In pigs, *Taenia solium* cysticerci are more commonly found in muscle tissue than in the brain (193). The opposite appears to be true in humans where cysticerci have a particular tissue tropism for the central nervous system. Why this is the case is not well understood. One possibility is that *Taenia solium* have not evolved to exist in humans as an intermediate host. Therefore, they require the relative immune privilege of the central nervous system to sufficiently evade the host immune response and maintain viability. Whilst *Taenia solium* cysticerci are found within the brain parenchyma, given their size, it is unlikely that the activated ova or cysticerci actively cross the blood brain barrier. Rather it is likely that the nascent cysticerci lodge in terminal arterioles or cerebral capillary beds where they then grow. Over time, and particularly following a host inflammatory response to the cysticerci, the blood brain barrier may break down (194).

#### Interplay Between the Parasite and the Immune System in the CNS

Following initial infection of the brain parenchyma by *T. solium* cysticerci, there is usually a lengthy period of several months to years in which the host shows minimal to no immune or inflammatory response and experiences no symptoms (195). This phase is termed the vesicular phase as the viable larvae appear as translucent, fluid filled vesicles or cysts. The cysticerci can utilize several mechanisms to evade or downregulate both the humoral and cellular arms of the host immune response (180). Modulation of the humoral immune response occurs in several ways including by taking up host immunoglobulins (IgG, IgM, IgE and IgG) in the cyst tegument to mask parasite antigens (196) and by releasing molecules (such as taeniastatin), which inhibit the complement pathway (197). Taenia cysticerci also modulate cells of the immune system in multiple ways. Firstly, they impede dendritic cell maturation (198), impair classical Toll-like receptor 4 (TLR4) mediated activation of microglia, macrophages and dendritic cells (199) and instead lead to alternative activation and the production of immunosuppressive cytokine such as TGF-ß and IL-10 (200–202). Furthermore, viable cysts can induce regulatory T-cell (T-regs) activity. Broadly speaking, viable, vesicular Taenia larvae are able to shift an initial, transient T-helper type 1 immune response toward a T-helper type 2 response, which is more permissive for chronic infection (199).

At some point, the cysts lose their ability to control the host immune response and begin to degenerate. The cyst wall and fluid become infiltrated by host inflammatory cells and the cysts become opaquer in appearance with turbid vesicular fluid. This is referred to as the colloidal phase and is associated with an intense T-helper type 1 inflammatory response (203). Following this phase, the cyst cavity starts to collapse, and the cyst becomes encompassed by host fibrotic tissue. This is termed the granular-nodular phase. Here the host inflammatory response reflects a more chronic phenotype featuring mixed T-helper type 1 and T-helper type 2 features (204). When imaged using CT scans, *T. solium* cysts in the colloidal or granular-nodular stage are accompanied by two neuroimaging features reflecting the presence of a host inflammatory response: ring-enhancement and visible perilesional oedema (200). Over time the cyst becomes entirely infiltrated by connective tissue, which may include accompanying calcium deposition. This calcific stage (180), is not accompanied by neuroimaging features reflective of a host inflammatory response (200). The colloidal, granular-nodular, and calcific stages of the cysts reflect dying or dead larvae, which are no longer viable.

#### Manifestations of Neurological Disease

As described above, seizures are the most common manifestation of parenchymal disease. However, when cysts occur in the ventricular and subarachnoid space headaches, intracranial hypertension, hydrocephalus and meningitis may occur (188). There is some correspondence between the likelihood of seizure occurrence and the preponderant stage of cysts in the brain. Seizures are typically infrequent when *T. solium* cysticerci are viable, and are most common when the cyst is dying or degenerating, and somewhat less common when the cysts are in the calcific stage (205). In general, seizures during all cyst stages are usually associated with a detectable inflammatory host immune response surrounding the cyst. This has led to the widely held view that seizures result, at least in part, from the host inflammatory response to the cyst (195, 206).

### Value of Neuroimmune Changes in Diagnostics and Therapies

The fact that viable cysts can suppress a host immune response and remain non-symptomatic makes diagnosis difficult in those with viable cysts and latent disease. Even in those with symptomatic NCC, diagnosis is notoriously challenging given the multitude of possible causes of seizures and the fact that expensive neuroimaging (CT or MRI scans) is not typically available in many endemic areas. Serology is certainly of diagnostic assistance with the most sensitive and specific test being the enzyme-linked immuno- electrotransfer blot (EITB) assay, which uses targeted antigens to detect antibodies to *T. solium* in patient serum. On this note (207) ﻿established a set of diagnostic criteria for NCC, which combines aspects of clinical history, neuroimaging and immunological evidence, as well as epidemiological factors, to form definitive guidelines for the diagnosis of NCC. This approach allows for a diagnosis to be made when some diagnostic modalities are not available.

An understanding of the interaction between parasite and host immune response, and particularly how this relates to symptom onset (i.e., seizures), is important for optimal management of NCC. Treatment must consider the location, viability, and number of the cysts as well as a characterization of the existing immune response to tailor the management plan to the individual concerned. Given that seizure severity is often correlated with dead or dying cysts and the accompanying immune response, the use of antiparasitic (cysticidal) drugs such as albendazole or praziquantel must be used with caution, particularly when many viable cysts are present. ﻿It is possible that mass death of larvae within the CNS could trigger an extensive inflammatory response and worsening of symptoms (208). This is a particularly important issue when cysts are in a sub-arachnoid or ventricular location and treatment could worsen hydrocephalus and/or cause a rapid rise in intracranial pressure. Therefore, when neuroimaging and definitive diagnosis is not available, it may not be sensible to proceed with cysticidal therapy and patients should primarily be managed symptomatically. This should be an especially strong consideration as cysts can often resolve naturally (209). That said, studies have shown that antiparasitic drugs can help reduce symptoms and hasten the resolution of lesions identified by neuroimaging. In addition, both patients and clinicians are understandably hesitant to allow a live parasite to persist untreated in the brain. Clearly the appropriate use of cysticidal agents remains as an area requiring further study and consensus. Steroids are an important component of treatment where they reduce the inflammation, which occurs following the degradation of cysts. As a result, prednisolone or dexamethasone are typically used as adjuncts to cysticidal therapy where they should be administered prior to the cysticidal drugs and continued for approximately a week following the end of antiparasitic treatment (208). Antiepileptic agents are also typically used and are effective in controlling NCC-related seizures. Surgery is rarely needed and only indicated if cysts are in a location that precludes cysticidal treatment and there is an urgent need for intervention (210).

### Knowledge Gaps

It is important that we better define the extent of human and porcine cysticercosis and human taeniasis in Africa. Epidemiological studies elucidating the extent of the disease in many parts of Africa are either non-existent or out of date. Improved knowledge on NCC prevalence should then inform government and policy makers to improve sanitation and agricultural practices in the areas concerned. This could include vaccination of pigs. Representative animal model systems on NCC should also be developed and used. These could help elucidate some of the fundamental pathological mechanisms underlying NCC and its associated neurological sequalae (211). Finally, further progress is needed in the development of treatment strategies, particularly for viable parenchymal NCC. An ideal treatment regimen would both kill cysticerci and safely prevent adverse effects associated with larval death and the associated host immune response.

## Other Parasites

### Amoebic Encephalitis

Primary amoebic meningo-encephalitis (PAM) is rarely diagnosed in Africa. However, several reports indicate the presence of pathogenic free-living amoebas in water and environment (212–215). Reported cases and seroprevalence studies indicate the occurrence of infections with *Naegleria fowleri* (216, 217), which can enter the CNS *via* the olfactory nerve, *Acanthamoeba* spp. present in water (218) and *Balamuthia* (219). Given the severity of PAM and the lack of effective treatments, more investigations are needed to ascertain the prevalence in African countries.

### *Schistosoma* spp.

*Schistosoma* spp. such as *S. mansoni*, *S. hematobium* and *S. japonicum* are extracellular helminths that are pathogenic in humans. Schistosomiasis is a neglected tropical disease currently infecting more than 140 million persons, of which 90% of the burden is in the SSA region (220). The prevalence of schistosomiasis in SSA is high in endemic regions of some countries e.g., above 50% amongst school age children in some communities in Ethiopia (221), 40-44.1% in Nigeria (222, 223), 21.1% in Ghana (224), 10.05-26.8% in Zimbabwe (225, 226), while some countries such as Senegal have reported a decrease from 78% to about 11% in school age children over a 12-year schistosomiasis control program (227).

Praziquantel is used for both preventative chemotherapy and treating schistosomiasis. Untreated, chronic schistosomiasis is associated with anemia, stunting, and reduced physical and mental capacity (228).

The lifecycle of *Schistosoma* spp. includes an intermediate host (fresh-water snails) where infective larvae (cercariae) grow and when released into water from the snails attach to and penetrate the skin of the definitive human host and move into the vascular system as schistomula (229). After initially residing in the lungs, they spread into the intrahepatic branches of the portal vein, where they mature into schistosomes. Schistosomes migrate and reside in the mesenteric veins (*S. mansoni* and *S. japonicum*) or pelvic veins (*S. haematobium*), where females lay eggs, which are secreted in feces or urine (229). Inflammatory granulomas form around eggs trapped in tissues and organs, such as the liver, intestinal tissue, and bladder, and result in intestinal, hepato-splenic, or urogenital disease (229)

Cerebral schistosomiasis/neuroeschistosomiasis, although considered rare, can occur when the parasite or its eggs lodge within CNS vessels and elicit an immune reaction (230–233) resulting in neuroinflammation and neurological symptoms such as seizures, encephalopathy with headache, visual impairment, motor deficits, ataxia and paralysis (229). *Schistosoma* eggs may spread to the CNS, through the arterial system as emboli after crossing previously developed pulmonary shunts or anastomosis from veins to arteries or through retrograde venous flow (234). They are deposited in cerebral vessels as emboli. Cerebral disease is mostly produced by *S. japonicum*, because the eggs are smaller and rounder and can reach the brain (233, 235). On the other hand, S*. mansoni* and *S. haematobium* cause mainly a spinal cord disease because the eggs are larger and are mostly retained in vessels at a lower spinal level (233, 235). Adult worms can also migrate *via* vessels to reach meninges and the choroid plexus where they may shed a lot of eggs into the CNS parenchyma, and this is probably the main cause of symptomatic neuroschistosomiasis (230, 232–234).

*Schistosoma* eggs secrete antigens such as glycans and glycoproteins that elicit an immune response leading to granuloma formation (230, 233, 236). In both human cases with neuroschistosomiasis caused by *S. japonicum* and mice that were injected with *S. japonicum* eggs in the brain microglia/macrophages constituted the major components of the granulomas surrounding the eggs (237). Patients with spinal cord schistosomiasis have increased levels of IL-1beta, IL-4, IL-6 and IL-10 and low concentrations of TNF-α and IFN-γ in both CSF and serum (238). In *S. mansoni* infected mice astrogliosis and microgliosis (228) were observed, as well as elevated IL-10 levels and decreased TNF-α expression (239). Thus, neuroschistosomiasis elicit a Th2 immune response in both humans and animals.

### *Toxocara* spp.

*Toxocara* spp. such as *T. canis* and *T. cati* are gastrointestinal ascarid nematodes distributed worldwide and found in canids such as including dogs, foxes, wolves, jackals and coyotes, and felids such as domestic cats (definitive hosts) and can also cause infections in humans (considered paratenic hosts) (240, 241). Infected definitive hosts excrete eggs in the feces, which embryonate in the environment and become infective.

Human beings can accidentally ingest eggs containing infective third-stage larvae from contaminated food, soil, and water, and through direct contact with infected pets such as cats and dogs (240, 241). Ingested eggs develop and hatch into larvae in the small intestine, penetrate the intestinal wall and migrate to various tissues through the circulatory system, resulting in immune and inflammatory tissue reaction that can lead to symptoms such as fever, headaches, coughing, and abdominal or limb pains (240, 241). Most infections remain asymptomatic or mild, however the most common clinical manifestations are visceral larva migrans and ocular larva migrans (240–242).

Toxocara larvae can invade the brain, leading to neurotoxocariasis or cerebral toxocariasis, In the brain the larvae can cause eosinophilic meningitis, encephalomyelitis, cerebral vasculitis and epileptic seizures. In experimental animal models, the presence of *Toxocara* larvae in the brain increases the permeability of the blood-brain barrier, the expression of proinflammatory cytokines and iNOS, and astrogliosis leading to neuronal damage (243–245); Disturbances in the profile of neurotransmitters, such as GABA, glutamate, serotonin, dopamine, and noradrenaline, have also been reported (244, 245).

### *Paragonimus* spp.

*Paragonimus* spp. such as P. westermani, P. africanus, P. heterotremus, P. kellicotti, P. mexicanus, P. siamensis, P. skrjabini, P. skrjabini miyazakii, and P. uterobilateralis, are lung flukes (trematodes) that cause paragonimiasis, a rare zoonotic disease, when they infect humans (definitive hosts) who have eaten undercooked freshwater crayfish or crabs (the intermediate hosts) infected with encysted metacercariae (246, 247). Humans can also get infected after eating raw meat of other animals that are paratenic hosts of the worms (247).

Pulmonary disease is the most common manifestation of the disease. However, beside the lungs the worms can infect other organs including the brain resulting in cerebral paragonimiasis or neuroparagonimiasis, which accounts for less than 1% of symptomatic paragonimiasis (246–248). In the brain worms lay eggs, which elicit an immune reaction and are surrounded by granulomatous lesions that can be cystic or solid. Cerebral paragonimiasis can manifest as headache, dizziness, spastic hemiplegia, hemianopsia, hemiparesis, dysarthria, seizures, mental retardation, visual disturbances, or motor and sensory disturbances (246, 247)

### *Onchocerca* spp.

*Onchocerca* spp. comprise a group of filarial nematodes transmitted by blackflies of genera *Simulium* and *Culicoides*. They primarily infest hoofed mammals, but canids, felids, and humans are also infected (249). *Onchocerca volvulus* is the human pathogen and causes the disease onchocerciasis commonly referred to as “river blindness (250). It was initially described in 1875 by a British naval surgeon John O’Neill among individuals in West Africa (251). *O. volvulus* is endemic in 31 countries in Africa, Yemen, Venezuela, and Brazil. Globally, approximately, 218 million people are at risk of infection with over 95% of these located in Sub Saharan Africa (252, 253). The primary clinical manifestations of onchocerciasis include varying degrees of onchodermatitis (skin complications) (254) and keratitis (visual impairment) (255) which result from inflammatory responses caused by microfilaria death and/or Wolbachia spp. (endosymbiont bacteria of *O. volvulus*) derived products within the skin and ocular cavities. Other conditions associated with *Onchocerca* infection include lymph node changes, reproductive abnormalities (such as secondary amenorrhea, spontaneous abortion, and infertility). In addition, chronic infection may cause low body weight and diffuse musculoskeletal pain (250).

More recently, neurological manifestations have been proposed as an additional clinical consequence of *Onchocerca* infections (256, 257). Although mechanistic data is lacking, epidemiological evidence suggest a strong association between *O. volvulus* and brain disorders – epilepsy, nodding syndrome and Nakalanga dwarfism (258). These associations were demonstrated in a number of community-based surveys in different African countries, from which a meta‐analysis reported a 0.4% increase in the prevalence of epilepsy for each 10% increase in the prevalence of onchocerciasis (259). Furthermore, a study conducted in Cameroon showed a temporal relationship between onchocerciasis and epilepsy highlighting a dose-dependent effect between the density of microfilaria and the risk of developing epilepsy in childhood (260, 261). Similarly, studies from Uganda have shown a decrease in the prevalence of epilepsy with declining *Onchocerca* burdens (262, 263). Epidemiological studies of both nodding syndrome and Nakalanga dwarfism also report consistent associations with *Onchocerca* (264, 265). Based on these data the term Onchocerca associated epilepsy (OAE) was coined to describe this group of disorders (258).

The pathological mechanisms by which *Onchocerca* may led to neurological sequalae remain poorly understood and under investigation (266). Several hypotheses have however been proposed with conflicting results: 1) Direct Invasion of CNS by the parasite or pathogenic proteins/metabolites (267, 268), 2) An *O. volvulus*–induced immune response through an inflammatory process or auto antibodies against neuron surface proteins (269–272), 3) A *Wolbachia* spp., dependent pathway (273), and finally, a tauopathy, manifesting as aggregates of tau protein in the brain (274, 275).

### Other Cestodes

Apart from T. solium, other globally distributed cestodes that may infect the brain are also present in Africa (246). Infection occurs via larvae of the genera Spirometra and Sparganum, which cause sparganosis (276) and metacestodes of the genus Echinococcus, which cause echinococcosis also known as hydatid disease (277). Both sparganosis and echinococcosis are neglected food-borne zoonotic diseases caused by the ingestion of contaminated food or water. Humans are accidental intermediate hosts within whom several body tissues may be infested (246). Importantly, brain infection can occur causing neurological disease (278). However, both cerebral sparganosis and echinococcosis are considered rare with the exact epidemiological picture being unclear.

Cerebral sparganosis occurs when plerocercoid larvae (spargana) invade the CNS resulting in tissue damage. The main clinical manifestations include fever, headache, neck stiffness, paresthesia, and seizures. Further, patients may suffer visual and sensory impairment, in addition to motor weakness (279, 280). Additionally, cerebral hemorrhage may manifest (281). The pathological mechanisms of cerebral sparganosis involves the formation of granulomatous lesions or eosinophilic granulomas following worm migration and inflammation (276, 280).

Cerebral echinococcosis is caused particularly by E. granulosus and E. multilocularis, which are forms of Echinococcus able to infect humans. As is the case for T. solium, ingested oncospheres (eggs) migrate through the intestinal wall and pass into the portal system of infected humans. In Echinococcus these are largely entrapped within the liver. However, some occasionally pass from the systemic circulation into the brain parenchyma. Within brain tissue, cerebral hydatid cysts can grow asymptomatically over an extended period to large sizes, this is particularly the case in children. The main clinical features of patients with intracranial hydatid cysts include raised intracranial pressure, blindness, loss of consciousness, focal neurological deficits and seizures (277, 282).

## Concluding Remarks

Neurological disorders caused by parasites within Africa include epilepsy, sleeping disorders, hyperalgesia, hemiparesis, dementia, long-term neuro-disability, and cognitive impairments. These disorders are because of the parasites or their products such as eggs being within the CNS causing structural damage and/or eliciting an immunological response.

During CM *Plasmodium* parasites sequestered in the CNS within erythrocytes cause the production of proinflammatory cytokines, such as TNF-α, LTα, IFN-γ, IL-1α, and IL-1β, which contribute to the hyperinflammatory state of this neurological disorder. The second stage of HAT, when trypanosomes have invaded the CNS, is accompanied by increased levels of proinflammatory cytokines such TNF-α and IFN-γ, which probably play an important role in hyperalgesia, sleep disturbances and alteration in circadian rhythm, which are prominent neurological features of HAT. The increased levels of proinflammatory cytokines such TNF-α and IFN-γ during cerebral toxoplasmosis serves a role of controlling the parasite in the CNS. Unlike the other CNS parasitic infections mentioned above live *T. solium* parasites are more associated with dampening of the immune system during neurocysticercosis, however when they degenerate a mixed Th1 and Th2 immune reaction is observed, which coincide with the development of seizures.

The immune molecules expressed during CNS infections by parasites can be exploited for therapeutic purposes. Immune molecules such as CXCL8, IFN-γ-induced CXCL10, CXCL13 and IL-10 have come out as strong biomarkers for disease staging of HAT. Finding other molecules including IFN-γ-induced molecules, which facilitate T cells and parasite crossing of the BBB, could be important both to understand the pathophysiology of the individual disease and to find possible biomarkers for staging e.g., HAT. More studies are needed to characterize the intermediate stage of HAT and define what it means in terms of treatment and therapeutic outcomes. In addition, targeting the exacerbated proinflammatory immune reaction that occur during PTRE because of treatment of HAT with melarsoprol could reduce fatalities. Targeting immune molecules such as TNF-α during cerebral malaria have not improved clinical outcomes, suggesting that there is a need to understand more about the role of different immune molecules during CM, to effectively target them for therapeutic purposes.

In conclusion, the immune system plays an important role in the neuropathology and neurological manifestations of CNS parasitic infections. Understanding the neuroimmunology of these parasites is essential not only for understanding the pathophysiology of the diseases they cause but also for the identification of biomarkers and therapeutic modalities to manage these disorders.

## Author Contributions

All authors listed have made a substantial, direct, and intellectual contribution to the work, and approved it for publication.

## Funding

*RI* is supported by Medical Research Council (MRC) and the UK Department for International Development (DFID) under the MRC/DFID Concordat agreement through an African Research Leadership Award to Richard Idro and Kevin Marsh, grant number MR/M025489/1. *RO* is supported through the DELTAS Africa Initiative [DEL-15-003]. The DELTAS Africa Initiative is an independent funding scheme of the African Academy of Sciences (AAS)’s Alliance for Accelerating Excellence in Science in Africa (AESA) and supported by the New Partnership for Africa’s Development Planning and Coordinating Agency (NEPAD Agency) with funding from the Wellcome Trust [107769/Z/10/Z] and the UK government. *JR* is supported by the National Research Foundation of South Africa, a Wellcome Trust Seed Award (214042/Z/18/Z), the South African Medical Research Council and by the FLAIR Fellowship Programme (FLR\R1\190829): a partnership between the African Academy of Sciences and the Royal Society funded by the UK Government’s Global Challenges Research Fund. *AB* is supported by grants from the Swedish Research Council (Vetenskapsrådet, 2018–02411) and the Olle Engkvist Byggmästare Foundation (193-609). *WM* is supported by grants from Kuwait University, Research Sector, and some of the studies described in this review have been supported by grants from the US NIH/Fogarty (1R21NS064888-01A1), from the Wellcome Trust (WT089992MA) and the Swedish Research Council (04480).

## Conflict of Interest

The authors declare that the research was conducted in the absence of any commercial or financial relationships that could be construed as a potential conflict of interest.

## Publisher’s Note

All claims expressed in this article are solely those of the authors and do not necessarily represent those of their affiliated organizations, or those of the publisher, the editors and the reviewers. Any product that may be evaluated in this article, or claim that may be made by its manufacturer, is not guaranteed or endorsed by the publisher.
